# Healthcare Environments and Spatial Variability of Healthcare Associated Infection Risk: Cross-Sectional Surveys

**DOI:** 10.1371/journal.pone.0076249

**Published:** 2013-09-19

**Authors:** Jean Gaudart, Elaine Cloutman-Green, Serge Guillas, Nikki D’Arcy, John C. Hartley, Vanya Gant, Nigel Klein

**Affiliations:** 1 Aix-Marseille Univ, UMR912 SESSTIM (AMU, INSERM, IRD), Marseille, France; 2 University College, London, Department of Statistical Science, London, United Kingdom; 3 Great Ormond Street Hospital NHS Trust, Camelia Botnar Laboratories, Department of Microbiology, London, United Kingdom; 4 University College London Hospital, Department of Microbiology, London, United Kingdom; 5 University College, London, Institute of Child Health, Infectious Diseases and Microbiology Unit, London, United Kingdom; Kenya Medical Research Institute - Wellcome Trust Research Programme, Kenya

## Abstract

Prevalence of healthcare associated infections remains high in patients in intensive care units (ICU), estimated at 23.4% in 2011. It is important to reduce the overall risk while minimizing the cost and disruption to service provision by targeted infection control interventions. The aim of this study was to develop a monitoring tool to analyze the spatial variability of bacteriological contamination within the healthcare environment to assist in the planning of interventions. Within three cross-sectional surveys, in two ICU wards, air and surface samples from different heights and locations were analyzed. Surface sampling was carried out with tryptic Soy Agar contact plates and Total Viable Counts (TVC) were calculated at 48hrs (incubation at 37°C). TVCs were analyzed using Poisson Generalized Additive Mixed Model for surface type analysis, and for spatial analysis. Through three cross-sectional survey, 370 samples were collected. Contamination varied from place-to-place, height-to-height, and by surface type. Hard-to-reach surfaces, such as bed wheels and floor area under beds, were generally more contaminated, but the height level at which maximal TVCs were found changed between cross-sectional surveys. Bedside locations and bed occupation were risk factors for contamination. Air sampling identified clusters of contamination around the nursing station and surface sampling identified contamination clusters at numerous bed locations. By investigating dynamic hospital wards, the methodology employed in this study will be useful to monitor contamination variability within the healthcare environment and should help to assist in the planning of interventions.

## Introduction

Notable progress has been made in the last 15 years in developing and implementing systems to reduce the risk of healthcare associated infections (HCAI) but with only a moderate reduction on the overall prevalence of HCAI in England from 8.2% (2006) to 6.4% in 2011 [1]. In the same period, HCAI has increased in patients in intensive care units (ICU) (23.4% in 2011). Some infections, such as *Staphylococcus* *sp.* and *Enterobacteriaceae* remain problematic within England representing 21.3% of the reported HCAI and 32.4% respectively [1]. Similar rates are seen worldwide e.g. Brazil (12.6% [2]), or Estonia (26% [2]) clearly demonstrating that HCAI rates are a continuing concern internationally [3,4,5].

The reasons why HCAI remain high in the face of universal infection control precautions may be because of the demanding environments required for patients with severe and complex pathologies, such as in ICUs and in facilities caring for high densities of immunocompromised patients. It is becoming increasingly apparent that the environment itself can be an important intermediary reservoir for potentially pathogenic microbes [6]. Surfaces, ward design, hand washing, staff behaviours, and ward management all contribute to pathogen behaviour [7] and the risk of HCAI [8]. How best to monitor and manage these environments is, however, still controversial [9,10].

If the spatial variability of microbiological contamination can be effectively evaluated, it could facilitate targeted infection control interventions to reduce the role of the environment as an intermediary source of cross transmission. The objective of this study was to develop a methodological approach to assess the spatial and temporal variability of bacteriologic contamination, both distant to and at bedsides in an ICU setting. By investigating a dynamic hospital ward, such an approach aimed to identify area of high levels of consistent contamination.

## Methods

### Data Collection

**Figure 1:**
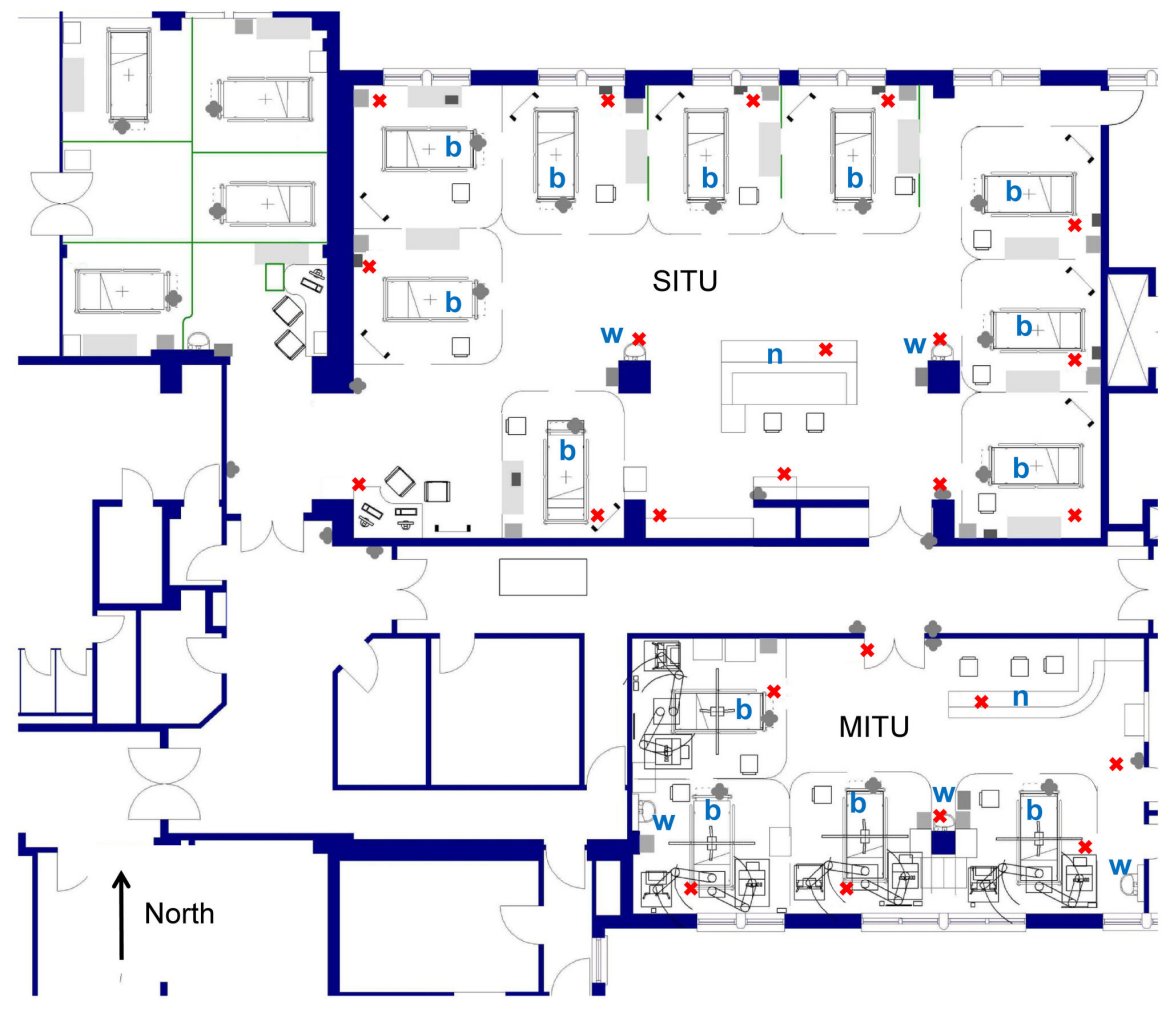
Map of the studied units. The red crosses represent the sampling locations. MITU: medical intensive care unit; SITU: surgical intensive care unit; b: bed; w: clinical waste bin; n: nurse’s station.

The screening procedure included 24 different sampling locations (five samples at each location), 8 in a four bedded medical intensive care unit (MITU) and 16 in a nine bedded surgical intensive care unit (SITU) (Figure 1) at the University College London Hospital (UCLH). Ward sampling was carried out with Tryptic Soy Agar (TSA) contact plates (5.5cm diameter i.e. 24cm^2^) in order to provide a quantitative measure on a non-selective growth medium, which would enable growth of skin and environmental flora [11,12,13]. Surfaces were sampled at these different locations in each bed space and distant to bed (door pressure panels or handles, nursing station etc). Samples were always taken from the same site on all locations (e.g. the centre panels of doors, the centre of the floor space, the centre of bed rails, centre of the bottom left bed wheel, above the bed head). Sites varied slightly depending on what furniture occupied the bed space at the time. Surface sampling included surfaces at different heights, low (<0.6 m), medium (0.6 to 1.2 m, including high touch surfaces) and high (>1.2 m). Air samples were taken from MITU and SITU in each bed space at the back right hand corner, around the nurse’s station and at access points onto the units. Air sampling was performed using a Sampl’air lite (Aes Chemunex), sampling 1m^3^ of air onto a blood agar plate [14]. Air sampling occurred at similar times to the surface sampling, separated in time by at least 1 hour to minimize user contamination. All plates were read and colony forming units (CFU) were counted per plate (5.5cm diameter, i.e. 24cm^2^) giving Total Viable Counts (TVC) that was recorded at 48hrs after incubation at 37°C [11].

In order to evaluate changes through time, the screening procedure (surface and air sampling) was replicated through three cross-sectional surveys at different times over a period of three months. The interval between each cross-sectional survey was of 1 month: it allowed comparison between the short stay SITU and the longer stay MITU as patient length of stay in SITU was less than a month whereas patient length of stay in MITU was over a month.

As many factors are involved in spatial distribution of bacterial contamination, the screening procedure also took into account some of these factors. Firstly, we recorded whether the bed was occupied or unoccupied and if the bedridden patient had been ascribed an infection control alert. Secondly, in each sampling location, surface types were selected for a different surface type and material at each location. Surface types included bed rails, floor, alcohol hand gel pump, bedside table, bed wheels, chair, clinical waste bin, storage trolley and unit top and shelf, top of computer, and surface material (porous and non-porous) are described in Table S1. Thirdly, samples were taken at the same time at each cross-sectional survey, without alteration to ventilation or cleaning regime, in order to ensure data comparability. Cleaning procedure was undertaken adhering to Department of Health Guidelines [15]: at UCLH, routine cleaning was undertaken using water and microfibre with no detergent, twice a day (8 am and 3 pm). Samples were taken three hours after routine morning cleaning in an attempt to standardize procedures. In addition, ITU cleaning does take place throughout the day as nurses actively wipe down surfaces with alcohol within bed spaces. The area studied had windows that did not open and air conditioning.

### Statistical analysis

#### Common specifications

In order to compare the different risk factors (type of surfaces, furniture, bedside, bed occupancy, height level and locations) involved in HCAI, TVC was analyzed using Generalized Additive Mixed Model (GAMM) [16,17]. This regressive approach was allowed to model the counts of micro-organisms growing on TSA plates, with a Poisson distribution model (using the log canonical link), adjusted on risk factors. The model selection was based on analysis of covariance for nested models and the Un-Biased Risk Estimator (UBRE) score. Diagnostic plots were examined to assess the quality of the model fit, according to Augustin et al. [18]. For each factor, incidence ratios (IRs) were calculated as the exponential of retrieved parameter estimates, comparing each class to the reference class. With an incidence ratio significantly higher than 1, a surface type was considered to be at risk of being more contaminated than the reference class, with a higher TVC, whatever the TVC of the reference class. A contrario, with an incidence ratio significantly lower than 1, a surface type was considered to have a lower TVC than the reference class. The statistical analysis was performed using the software R 2.10.1 (The R Foundation for statistical computing, Vienna, Austria), and the mgcv 1.7-22 package developed by Simon Wood [19]. Maps were performed using the geographic information system ArcGIS (Environmental Systems Research Institute, Redlands, California). All p-values were compared to the classical α-threshold of 0.05.

#### GAMM for surface types

Associations between bacterial counts and surface types were assessed using a GAMM model including the following risk factors: 'Surface types' and 'Ward' (MITU or SITU). The 'Surface types' variable had eleven classes, which were bed rails (reference class), floor, alcohol hand gel pump, bedside table, bed wheels, chair, clinical waste bin, storage trolley and unit top and shelf, top of computer. For the variable 'Ward', the reference class was MITU. The location according to beds and bed occupancy (non-bedside, unoccupied bedside, occupied bedside) and the cross-sectional survey date were modelled as random effect. In addition, a comparison of TVC between porous and non-porous materials and between different materials was provided by using Kruskall-Wallis ranking test.

#### Spatial GAMM

Spatial analysis was performed to assess the spatial variability of micro-organisms in the air and on surfaces using GAMM. Because numerous surface samples were taken at each location, mean TVCs were used. The risk factors, included in the regression model were: 'Bedside' including occupancy of beds (non bedside, unoccupied bedside, occupied bedside), 'Ward' (MITU and SITU), 'Height level' (<0.6m, [0.6m-1.2m], >1.2m) and locations. The reference classes were respectively non bedside, MITU, height level <0.6m. The locations of each sample were referenced using Cartesian coordinates, which where modelled using thin plate splines [20]. The selected models were also used for mapping the TVCs from the surface environment and from the air environment, using gridded coordinates. Bedside was specified according to the distance to a bed, and the occupancy was specified according to the current occupancy for each cross-sectional survey.

## Results

TVCs were obtained for a total of 370 samples, i.e. between 120 and 130 samples for each cross-sectional survey. During the first and second cross-sectional surveys, all of the bed spaces on MITU (100%) and 5 of the 9 beds on SITU (55,6%) were occupied. During the third cross-sectional survey, 3 of the 4 beds on MITU (75%) and 6 of the 9 beds on SITU (66.7%) were occupied. Organisms were identified to genus level, and the predominant genus identified was *Staphylococcus* .

Crude counts are presented in Table 1 and Incidence Ratios in Tables 2, 3, 4. The lower height level (<0.6m) was consistently the most contaminated within the SITU (median TVCs between the first, second and third cross-sectional surveys were 182, 107 and 350 per 24cm^2^ respectively). In contrast the pattern of contamination in MITU was more variable between cross-sectional surveys. For example the lower height level (<0.6m) was most contaminated for the second cross-sectional survey (median TVC 350 per 24cm^2^), but with the highest level (>1.2m) most contaminated for the first and third cross-sectional surveys (median TVCs 200 and 350 per 24cm^2^ respectively). Mid-level surfaces sampled (between 0.6 and 1.2m) did however demonstrate consistently lower levels of contamination during all the three cross-sectional surveys (median TVCs 34.4, 28.7, 55.4 per 24cm^2^ respectively).

**Table 1:** Total viable counts (TVC) per 24cm^2^ plates (5.5cm diameter).

Care unit	Height level	First cross-sectional survey	Second cross-sectional survey	Third cross-sectional survey
SITU	Level 0	182 (n=15)	107 (n=16)	350 (n=16)
		(40-350)	(17-350)	(18-350)
	Level 1	71.2 (n=33)	34.8 (n=36)	64.5 (n=36)
		(2-269)	(1.5-350)	(3-350)
	Level 2	79.2 (n=13)	29.5 (n=15)	14.5 (n=15)
		(5-350)	(2.5-197)	(1-350)
	Air	98.5 (n=12)	60 (n=11)	164 (n=11)
		(60-167)	(27-158)	(39-419)
MITU	Level 0	150.3 (n=7)	350 (n=9)	205 (n=9)
		(6-623)	(2-350)	(4-350)
	Level 1	34.4 (n=24)	28.7 (n=25)	55.4 (n=25)
		(12.3-238.3)	(2-126)	(9.7-350)
	Level 2	200 (n=5)	27 (n=5)	350 (n=5)
		(27-350)	(6-350)	(22-350)
	Air	103 (n=7)	88 (n=7)	182 (n=7)
		(99-217)	(50-167)	(51-213)

Median, sample size (n) and (min, max), are presented for each cross-sectional survey, at each height level and care unit.

Level 0: <0.6m; Level 1: [0.6m-1.2m]; Level 2: >1.2m

SITU: surgical intense care unit MITU: medical intensive care unit

**Table 2:** Spatial analysis of Air samples.

Cross-sectional surveys	Cofactors		IR [CI95%]	p-value
(% explained deviance -n^§^)				
First cross-sectional survey				
(81.5% - n=19)	Bedside	Non Bedside*	1	-
		Non occupied Bedside	0.74[0.58;0.93]	0.007**
		Occupied Bedside	1.11[0.91;1.35]	0.26
	Ward	MITU*	1	-
		SITU	0.33[0.2;0.57]	<0.001**
	Spatial location***		-	<0.001**
Second cross-sectional survey				
(85.7% - n=19)	Bedside	Non Bedside*	1	-
		Non occupied Bedside	0.71 [0.52;0.97]	0.02**
		Occupied Bedside	0.6[0.47;0.76]	<0.001**
	Ward	MITU*	1	-
		SITU	14.34 [8.08;25.44]	<0.001**
	Spatial location***		-	<0.001**
Third cross-sectional survey				
(49.6% - n=19)	Bedside	Non Bedside*	1	-
		Non occupied Bedside	1.92 [1.54;2.39]	<0.001**
		Occupied Bedside	1.96 [1.66; 2.31]	<0.001**
	Ward	MITU*	1	-
		SITU	0.07 [0.04;0.11]	<0.001**
	Spatial location***		-	<0.001**

Risk factors were assessed each day by using Generalized Additive Mixed Model, adjusted on Bedside (occupied or not), Ward and location.

§ n: number of locations

* reference class

** p<0.05

*** spatial location was modelled by thin plate splines not providing unique IR.

IR: incidence ratio SITU: surgical intensive care unit MITU: medical intensive care unit

**Table 3:** Spatial analysis of Surface samples.

Cross-sectional surveys	Cofactors		IR [CI95%]	p-value
(% explained deviance -n^§^)				
First Cross-sectional survey				
(38% - n=49)	Bedside	Non Bedside*	1	-
		Non occupied Bedside	0.78 [0.67;0.89]	0.0002**
		Occupied Bedside	1.72 [1.54;1.91]	<0.001**
	Ward	MITU*	1	-
		SITU	0.77[0.59;1]	0.05**
	Height	Level 0 <0.6m	1	-
		Level 1 [0.6-1.2m]	0.39 [0.37;0.42]	<0.001**
		Level 3 >1.2m	0.59 [0.56;0.64]	<0.001**
	Spatial location***		-	<0.001**
Second cross-sectional survey				
(56% - n=57)	Bedside	Non Bedside*	1	-
		Non occupied Bedside	6.96 [5.54;8.73]	<0.001**
		Occupied Bedside	3.14 [2.84;3.48]	<0.001**
	Ward	MITU*	1	-
		SITU	0.18 [0.13;0.24]	<0.001**
	Height	Level 0 <0.6m *	1	-
		Level 1 [0.6-1.2m]	0.39 [0.37;0.42]	<0.001**
		Level 3 >1.2m	0.31 [0.29;0.33]	<0.001**
	Spatial location***		-	<0.001**
Third cross-sectional survey				
(27% - n=57)	Bedside	Non Bedside*	1	-
		Non occupied Bedside	2.24 [2.04;2.46]	<0.001**
		Occupied Bedside	1.5 [1.39;1.61]	<0.001**
	Ward	MITU*	1	-
		SITU	0.09 [0.07;0.12]	<0.001**
	Height	Level 0 <0.6m *	1	-
		Level 1 [0.6-1.2m]	0.47 [0.44;0.49]	<0.001**
		Level 3 >1.2m	0.6 [0.57;0.64]	<0.001**
	Spatial location***		-	<0.001**

Risk factors were assessed each day by using Generalized Additive Mixed Model, adjusted on Bedside (occupied or not), Ward, height and location.

§ n: number of locations

* reference class

** p<0.05

*** spatial location was modelled by thin plate splines not providing unique IR.

IR: incidence ratio SITU: surgical intensive care unit MITU: medical intensive care unit

**Table 4:** Effects on bacterial counts for the different surface types sampled.

Cofactors		IR [CI95%]	p-value
Surface types	Bed rails*	1	-
	Floor	1.18 [0.76;1.83]	0.46
	Alcohol hand gel pump	0.27 [0.1;0.79]	0.02**
	Bed side table	0.087 [0.01;0.74]	0.03**
	Bed wheels	1.97 [1.21;3.21]	0.01**
	Chair (seat)	0.45 [0.09;2.24]	0.32
	Clinical waste bin	0.61 [0.29;1.28]	0.19
	Storage trolley	0.41 [0.18;0.91]	0.03**
	Storage unit - shelf	0.62 [0.21;1.84]	0.39
	Storage unit - top	0.48 [0.25;0.89]	0.02**
	Top of computer	1.06 [0.61;1.84]	0.83
Ward	MITU*	1	-
	SITU	0.89 [0.64;1.24]	0.49

The adjusted incidence ratios (IR) are presented with their 95% confidence intervals.

* reference class

** p<0.05

SITU: surgical intensive care unit MITU: medical intensive care unit

To enable the impact of location and bed occupancy on contamination to be assessed, TVCs were analysed in a statistical model as described in the methods. As Tables 2-4 demonstrate, there was a highly significant variation in contamination throughout the units analysed. When TVCs were adjusted for ward (SITU or MITU), bed occupancy and sample location, mid and high-level surface samples were significantly less contaminated than samples taken from locations under 0.6 m (Incidence Ratios -IR- respectively at 0.39, 95% Confidence interval [0.37; 0.42], and 0.59 [0.56; 0.64] (first cross-sectional survey), 0.39 [0.37; 0.42] and 0.31 [0.29; 0.33] (second cross-sectional survey) and 0.47 [0.44; 0.49] and 0.6 [0.57; 0.64] (third cross-sectional survey)).

Levels of contamination on the surface types studied are displayed in Table 4. Contamination is presented as adjusted incidence ratios (IR) related to TVC on the bed rails (reference class). Bed wheels, bedside table, storage trolley, alcohol hand gel pump, and top of the storage unit, were all more contaminated than bed rails. Bed wheels were most contaminated with a mean TVC 1.97 times higher than on the bed rail (95% Confidence Interval 95%CI [1.12; 3.21]). Alcohol hand gel (wall mounted) and alcohol hand gel pump (patient bed side), (IR=0.27 [0.1; 0.79]), bedside table (IR=0.087 [0.01; 0.74]), storage trolley (IR=0.41 [0.18; 0.91]) and the top of the storage unit (IR=0.62 [0.25; 0.89]) were all less contaminated than bed rails. The comparison of TVC between porous and non-porous materials and between different materials showed no significant differences (p=0.53 and p=0.198, respectively).

Surfaces located at occupied bedsides were always more contaminated than surfaces located away from bed spaces (IR at 1.72, [1.54; 1.91] -first cross-sectional survey-, 3.14 [2.84; 3.48] -second cross-sectional survey- and 1.5 [1.39; 1.61] -third cross-sectional survey). Apart from the first cross-sectional survey, surfaces located at unoccupied bedsides were also more contaminated (IR at 0.78, [0.87; 0.89] -first cross-sectional survey-, 6.96 [5.54; 8.73] -second cross-sectional survey- and 2.24 [2.04; 2.46] -third cross-sectional survey). Surfaces sampled at SITU were always significantly less contaminated than surfaces sampled at MITU (IR at 0.77 [0.59; 0.99] -first cross-sectional survey-, 0.18 [0.13; 0.24] -second cross-sectional survey-, and 0.09 [0.07; 0.12] - third cross-sectional survey).

**Figure 2:**
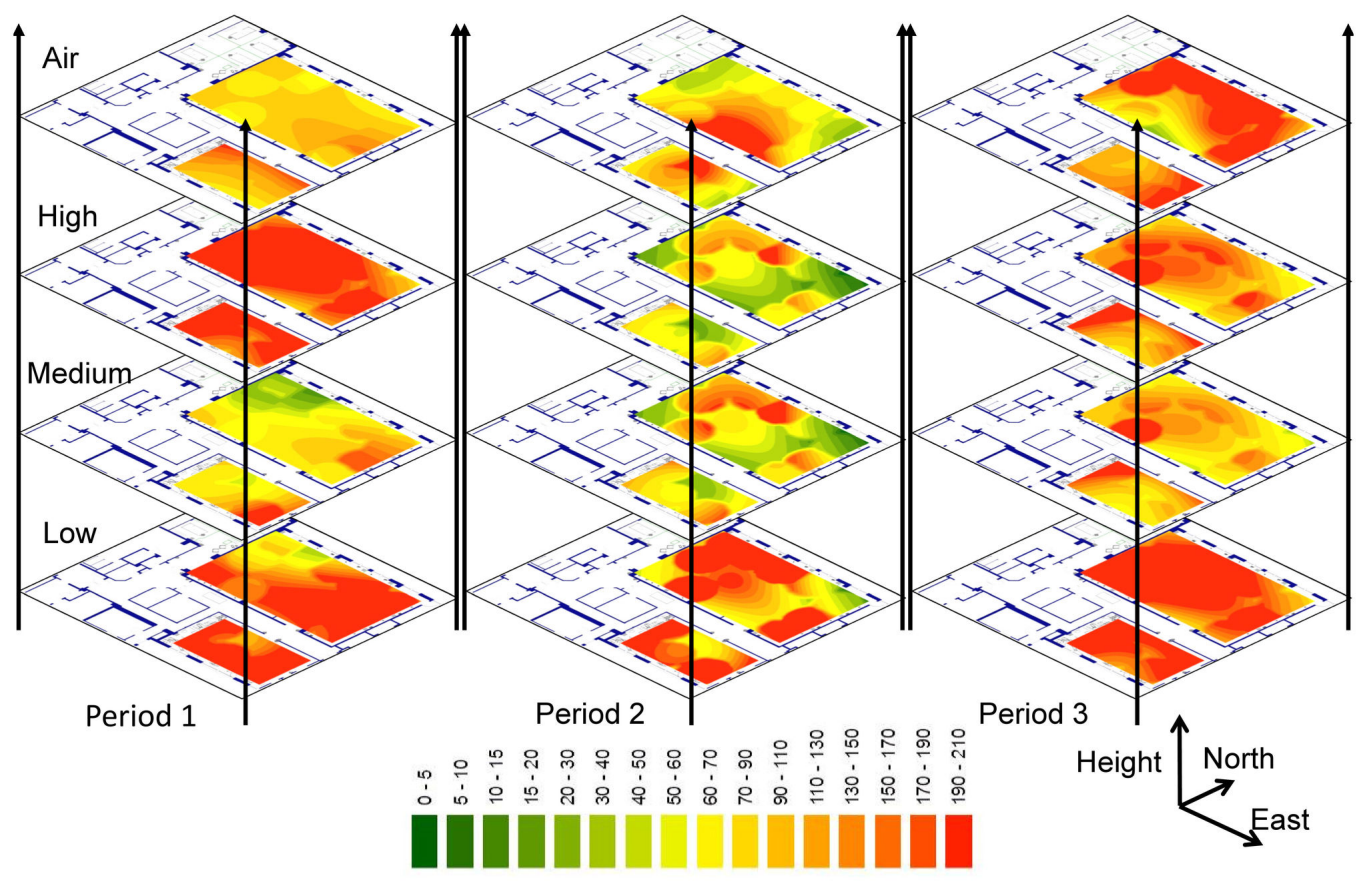
Estimation of the counts of micro-organisms. Results were adjusted on bedside, bed occupancy, height level (for surface analysis), Ward and location. Total Viable Count (TVC) estimations for the three cross-sectional surveys at the different height levels including air sampling are presented at each location. The coloured scale showed the values of TVC.

Air contamination was variable, showing less contamination at unoccupied bedsides during the first cross-sectional survey, a cluster around the nurse’s station during the second cross-sectional survey, and more contamination at bedsides during the third cross-sectional survey (air samples IRs at unoccupied and occupied bedsides were 0.74 [0.58; 0.93] and 1.11 [0.91; 1.35] respectively -first cross-sectional survey-, 0.71 [0.52; 0.97] and 0.6 [0.47; 0.76] respectively -second cross-sectional survey-, and 1.92 [1.54; 2.39] and 1.96[1.66; 2.31] respectively -third cross-sectional survey). Figure 2 shows clusters of contamination predominantly around bed locations but, during the second cross-sectional survey, in the air around the nurse’s station.

The regression models developed for this study, which utilise TVC data, location, and occupancy, were found to be accurate at the first and second cross-sectional surveys (with percentages of explained deviance at 81,5 and 85,7% respectively). However these models were less accurate for the third cross-sectional survey. Sample location remained a significant factor in predicting colony counts (p<0.0001) for all cross-sectional surveys.

## Discussion

In this study we have analyzed the environmental variability of micro-organisms within an ICU healthcare environment, by measuring TVCs on surfaces and in air. We aimed to establish an approach to facilitate monitoring and analysis of microbial contamination, which could be applied to healthcare settings, even if our approach did not evaluate precisely all the factors involved in the contamination variability (such as healthcare worker behaviour, patient status, modification of air-conditioning, people-traffic). The results showed that, for this particular environment, while contamination relationships were complex, some patterns emerged that could be modelled and used to estimate the distribution of microbial contamination. In this particular setting hospital design per se could not be the sole determinant of contamination. Staff behaviours, cleaning procedures and the nature and severity of a patient’s condition may also have been important contributors to levels of bacteria. Such factors are amenable to mitigation by changes in ward layout to influence staff behaviour, improving accessibility to cleaning staff and by changes in healthcare components design such as use of easy clean surfaces.

Occupation of bed spaces and illness severity appeared to be consistent predictors of contamination. Air and surface TVCs were generally higher in MITU where patients are usually long stay and require care, such as feeding, in which there is substantial interaction with their bedside environment and particularly with relatives. These long-stay patients have more of their own possessions within the bed space and visitation is encouraged, tending to be regular and prolonged. SITU patients are usually short-stay, ventilated and require high intensity support from staff. Visitation is limited to 2 people per bedside and is only allowed during certain hours. Visitors, due to the severity of illness on this ward, have limited interaction with patients and the bedside environment. Prolonged stay in ICU has been shown to increase the risk of acquiring an HCAI [21].

Colony counts in air and on surfaces varied between locations, height, bedside location and bed occupancy. There were differences between air and surface samples, indicating that the source of microbes may differ. Air sampling provides both a measure of transient aero-contamination and a snap shot of more widespread microbial levels. Surface sampling is affected by aero-contamination, as some particles will eventually settle on surfaces. King et al. [22] showed that bioaerosols can be deposited across a room at different distances from a source, due to air movement, which can be modified by furniture and people behaviour. Organisms that have settled may then be transferred to other sites by touch, and by air eddy currents generated by human traffic. Surface samples are highly affected by human behaviours within the ward environment, and particularly by touch. Our results show that the air and surfaces within bed spaces were consistently highly contaminated. In contrast, the aero-contamination at the SITU nurse’s station during the second cross-sectional survey, occurred in the context of low surface TVCs. We also observed surfaces, which were heavily contaminated in areas of low aero-contamination. Surfaces within the middle height range were generally less contaminated. Even though the cleaning regime wasn’t comprehensively assessed, cleaning was probably also an important factor in determining microbial levels.

Our results, in combination with other studies [6,23,24], support continuous environmental monitoring and not only in response to outbreaks. Continuous monitoring will permit the establishment of baseline data for units that can be used to target interventions. Sampling will also permit the identification of surfaces that are linked with higher levels of contamination where a design solution may be sought. It is important not only to evaluate the hotspots within a ward, such as bed spaces and nurses stations, but also to evaluate more globally what the HCAI risks are of a 'functional unit'. Such data can be used to inform benchmarking as a means of evaluating cleaning regimes [25,26,27]. Numerous studies meticulously describe surface cleanliness as these relate different cleaning procedures, including detergent use, design, behaviour [12,28,29,30,31], and production and distribution of aerosols [13,32,33]. Our work did not evaluate cleaning procedures nor the factors involved in distribution of aerosols. ITU cleaning does take place throughout the day as nurses actively wipe down surfaces with alcohol within bed spaces. Therefore we cannot state with certainty what impact cleaning would have had on our results. It was not our intention to ascertain the quality of cleaning but to measure 'real life' microbial levels within a hospital environment. Better cleaning protocols and systems would doubtless have great impact at those sites identified as high risk. Our work does however provide information that could promote easier, more effective cleaning for less effort by better design and use of space.

HCAI has not been eliminated despite increased interventions [15,34,35] and it is not known which component of the control process is most effective nor which to pursue further. Levels of micro-organisms within the environment may be related to healthcare design and/or processes. A scientific approach to studying the relationship between healthcare design and HCAI risk has been problematic due to the multitude of factors that affect a patient’s interaction with both people and environment. Healthcare environments, hospital design and healthcare behaviours contribute to the risk of HCAI, in addition to the health status of each patient. These components include numerous variables including cleaning regimes, ventilation, bed locations and occupancy, windows and doors, material, staff and patient movements and height level. The complex interactions that do and can occur within the healthcare setting should be borne in mind when monitoring contamination. In this study we assessed the spatial variability of contamination, using a monitoring approach based on surface and air sampling at different location, height and time. Even when the main covariates were taken into account (distance to bed, bed-occupancy, care unit, location, height level, surface type), spatial variability still remained. This indicates that other cofactors were influencing contamination. This approach however can be used to assess the spatial variability of contamination over the working day, or before and after a specific intervention or event (e.g. an intervention in the ventilation system or a major contamination). By identifying areas of high levels of consistent contamination, the methodology employed in this study will be useful to monitor contamination variability within the healthcare environment and should help to assist in the planning of interventions.

## Supporting Information

Table S1 Sampled surface: type of material and porosity.
(DOCX)
