# Supplementary material for: Healthcare Environments and Spatial Variability of Healthcare Associated Infection Risk: Cross-Sectional Surveys
Source: PLoS One. 2013 Sep 19;8(9):e76249. doi: 10.1371/journal.pone.0076249 (PMC3777895; doi:10.1371/journal.pone.0076249)
Supplement: Table S1 — Sampled surface: type of material and porosity. (DOCX) [file pone.0076249.s001.docx]

Table S1

Sampled surface: type of material and porosity

| Surface | Material | Porosity |
| --- | --- | --- |
| Alcohol hand gel pump | Plastic | Non porous |
| Bed rails | Metal | Non porous |
| Bed side table | Plastic | Non porous |
| Bed wheels | Metal | Non porous |
| Chair (seat) | Fabric | Porous |
| Clinical waste bin | Metal | Non porous |
| Floor | Lino | Non porous |
| Storage trolley | Plastic | Non porous |
| Storage unit - shelf | Plastic | Non porous |
| Storage unit - top | Plastic | Non porous |
| Top of computer | Plastic | Non porous |
